# Excellent response of lung adenocarcinoma harboring a rare SLC8A1 downstream intergenic region ALK fusion to ceritinib treatment: A case report

**DOI:** 10.1097/MD.0000000000030255

**Published:** 2022-08-26

**Authors:** Lei Fang, Guozheng Ding, Muzi Wang, Yuanzi Ye, Xuebo Yan, Peishan Ding, Jiong Wang, Yanbei Zhang

**Affiliations:** a Department of Geriatric Respiratory and Critical Care, Provincial Key Laboratory of Molecular Medicine for Geriatric Disease, Anhui Geriatric Institute, the First Affiliated Hospital of Anhui Medical University, Hefei, Anhui, China; b Department of Pulmonary, Anqing Municipal Hospital, Anqing, Anhui, China; c Department of Pulmonary and Critical Care Medicine, the First Affiliated Hospital of University of Science and Technology of China (Anhui Provincial Hospital), Hefei, China; d Department of Pathology, the First Affiliated Hospital of Anhui Medical University, Hefei, Anhui, China.

**Keywords:** anaplastic lymphoma kinase, ceritinib, intergenic region, lung adenocarcinoma, solute carrier family 8 member A1

## Abstract

Anaplastic lymphoma kinase (ALK) gene fusion, an important driver gene alteration leading to the development of lung cancer, occurs in 5% of nonsmall cell lung cancer (NSCLC) cases in China. In addition to echinoderm microtubule-associated protein-like 4 (EML4)-ALK, which is the most common type of ALK fusion, various fusion partner genes have been identified in recent years. However, ALK intergenic breakpoint fusions confound fusion detection and targeted treatment.

**Patient concerns:**

A 40-year-old woman presented to our hospital with a 2-month history of a cough.

**Diagnosis:**

Based on the right hilar lymph node biopsy and positron emission tomography computed tomography (PET-CT) examination, the patient was diagnosed with “stage IV lung adenocarcinoma” showing metastases in the mediastina, right hilar lymph nodes, and C7 vertebral body. A rare solute carrier family 8 member A1 (SLC8A1) downstream intergenic region ALK fusion was identified in biopsy specimens using next-generation sequencing (NGS).

**Interventions:**

The patient received first-line molecular-targeted therapy (ceritinib).

**Outcomes:**

After nearly 9 months, the best evaluation of partial remission (PR) was obtained.

**Lessons:**

This is the first clinical evidence of advanced NSCLC due to a rare SLC8A1 downstream intergenic region ALK fusion that has been effectively treated with ceritinib. Whether this finding represents an inherent property of this fusion protein or its unique clinicopathological characteristics in patients carrying this fusion protein remains to be investigated. Moreover, the patient’s durable response to ceritinib and future resistance mechanisms require further follow-up.

## 1. Introduction

Lung cancer is the leading cause of cancer-related deaths, and its incidence and mortality are rapidly increasing. nonsmall cell lung cancer (NSCLC) accounts for approximately 85% of all lung carcinomas, and the 5-year survival rate is extremely low.^[1]^ With the development of next generation sequence (NGS), anaplastic lymphoma kinase (ALK) has been proven to be another main oncogene-driven gene after the epidermal growth factor receptor (EGFR), and a number of ALK fusion subtypes have been detected in recent years.^[2]^ In addition to the most common and classical rearrangement of echinoderm microtubule-associated protein-like 4 (EML4) and ALK, it harbors the 5’ end of EML4 fused to the entire ALK kinase domain and results in constitutive kinase activation.^[2]^ To date, at least 90 distinct non-EML4 ALK fusion partners have been identified in ALK + NSCLC. Furthermore, 28 potential fusion partners due to intergenic ALK rearrangements have been discovered.^[3]^ However, intergenic breakpoint fusions, in which 1 or both genomic breakpoints localize to intergenic regions, confound fusion detection and treatment.^[4]^ Here, we describe the discovery of a rare intergenic ALK fusion—solute carrier family 8 member A1 (SLC8A1)-ALK fusion in a Chinese female patient diagnosed with stage IV lung adenocarcinoma using NGS-based biopsy specimen profiling, as well as the first clinical evidence suggesting an excellent response to ceritinib via SLC8A1 downstream intergenic region ALK fusion in NSCLC.

## 2. Case presentation

**Figure 1.**
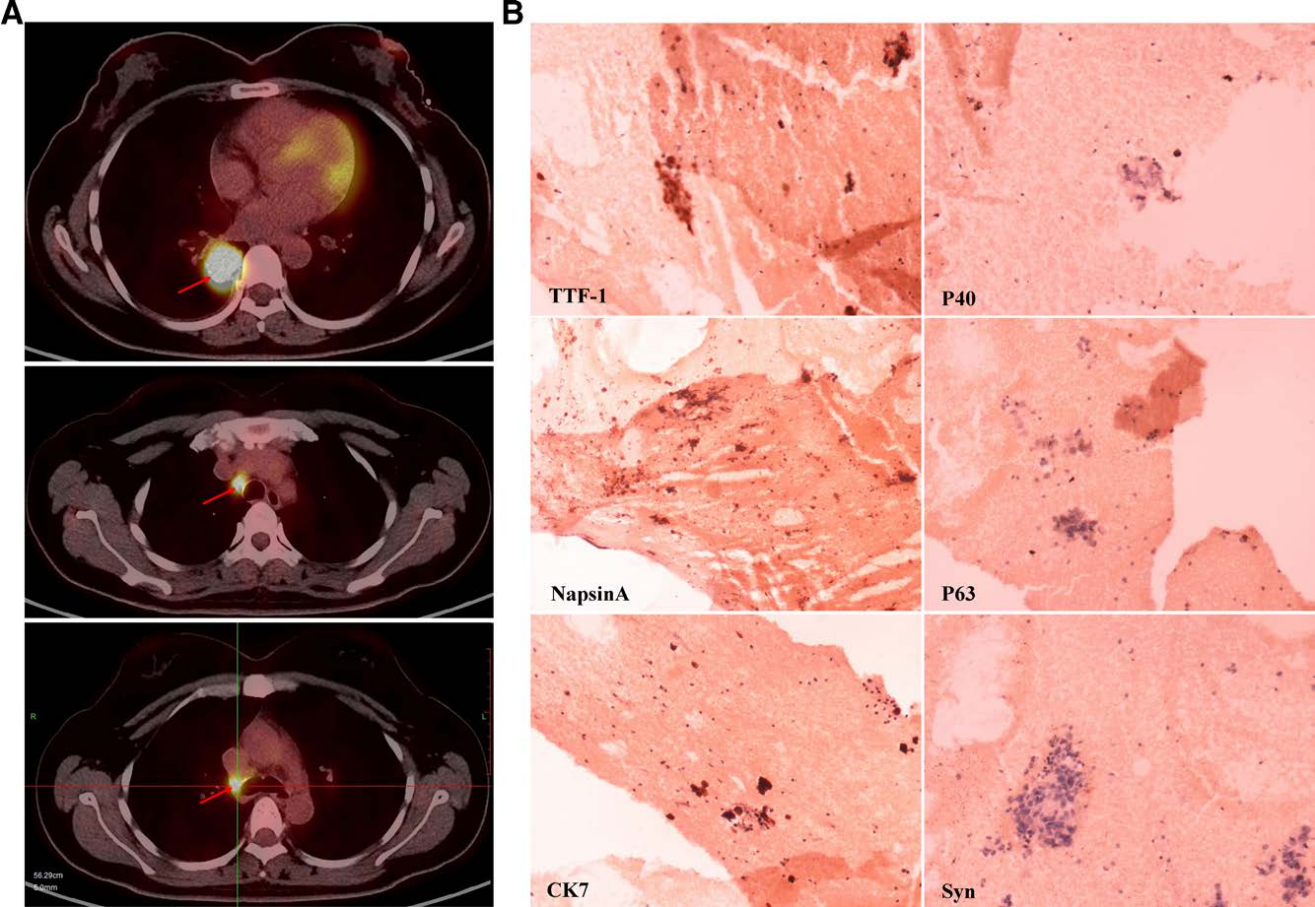
A Chinese female patient was diagnosed with stage IV lung adenocarcinoma. (A) Positron emission tomography computed tomography (PET-CT) indicated localized malignant lesions in the lower lobe of the right lung and mediastinal and right hilar lymph nodes. (B) Immunohistochemical (IHC) analysis showed that the tumor cells were positive for TTF-1, NapsinA and CK7 and negative for P40, P63, and Syn (×200).

A 40-year-old Chinese female nonsmoker with no personal history of heart disease, hypertension, diabetes mellitus, or cancer presented to our hospital with a 2-month history of cough. Chest computed tomography (CT) scan images showed a 2.6 cm mass in the lower lobe of the right lung and enlargement of mediastinal and right hilar lymph nodes (Fig. 3A); intense high-metabolic lesions in these regions were confirmed by examination with positron emission tomography computed tomography (PET-CT) (Fig. 1A). Right hilar lymph node biopsy was performed using endobronchial ultrasound-guided transbronchial needle aspiration (EBUS-TBNA) but not the primary lung mass due to its proximity to the heart. Immunohistochemical (IHC) analysis was positive for TTF-1, NapsinA, and CK7 and negative for P40, P63, and Syn (Fig. 1B). Based on the IHC analysis, the pathological diagnosis was lung adenocarcinoma. Interestingly, serum carcinoembryonic antigen (CEA) level was normal (Fig. 3H). PET-CT revealed a bone metastasis at the C7 vertebral body (Fig. 3F). Thus, the patient was diagnosed with stage IV lung adenocarcinoma with multiple metastases in the mediastina, right hilar lymph nodes, and C7 vertebral body in September 2020.

**Figure 2.**
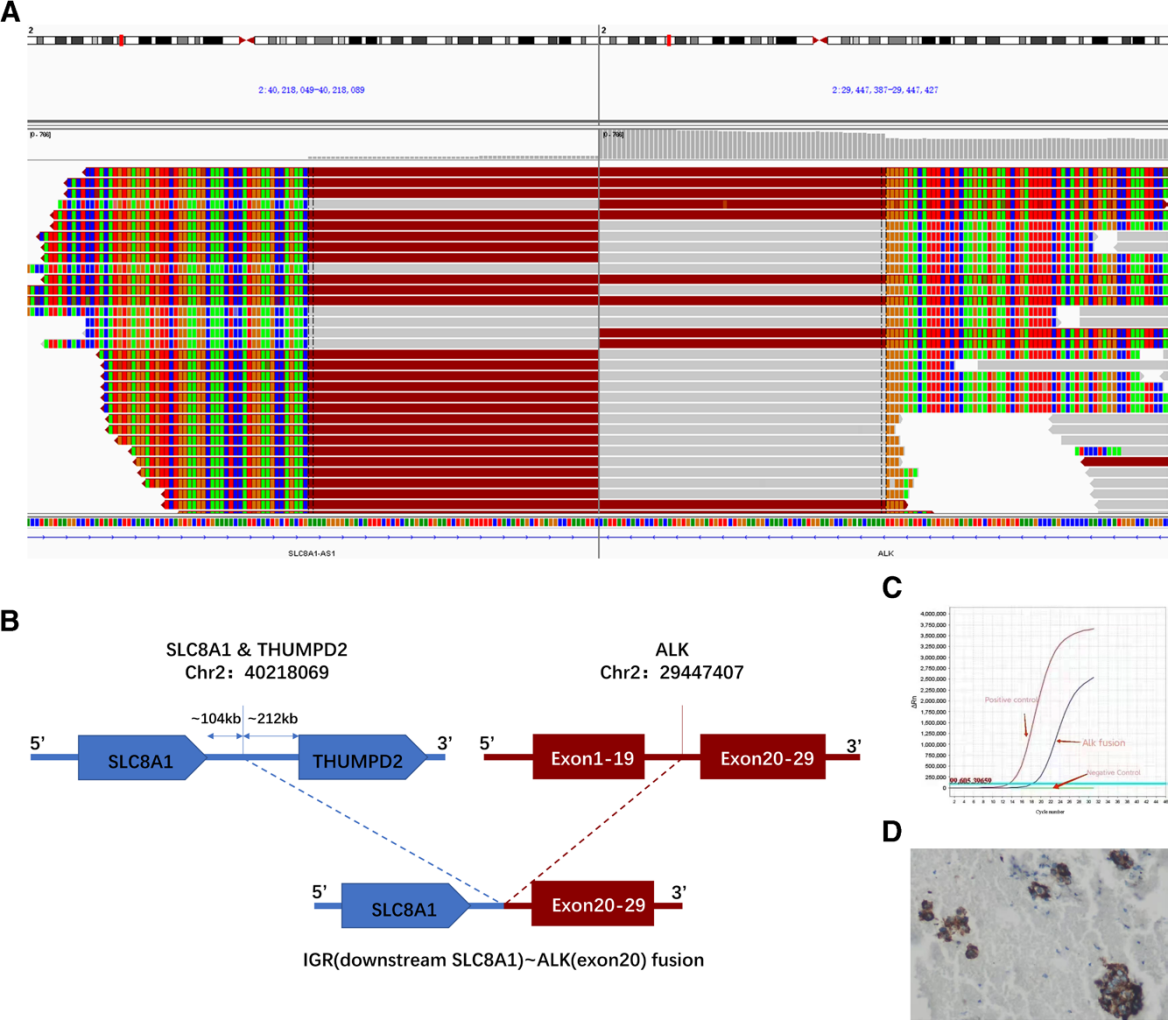
A rare SLC8A1 downstream intergenic region ALK fusion was discovered in the patient’s biopsy specimen. (A) Integrative Genomics Viewer (IGV) showed the SLC8A1-ALK intergenic fusion detected by capture-based next-generation sequencing (NGS) from right hilar lymph node metastasis formalin-fixed, paraffin-embedded clinical sample. (B) A diagram of the SLC8A1-ALK intergenic fusion. (C) Confirmation of the ALK fusion by real-time quantitative polymerase chain reaction (qPCR). (D) Immunohistochemistry (IHC) staining indicated a strong expression of ALK (D5F3 antibody).

**Figure 3.**
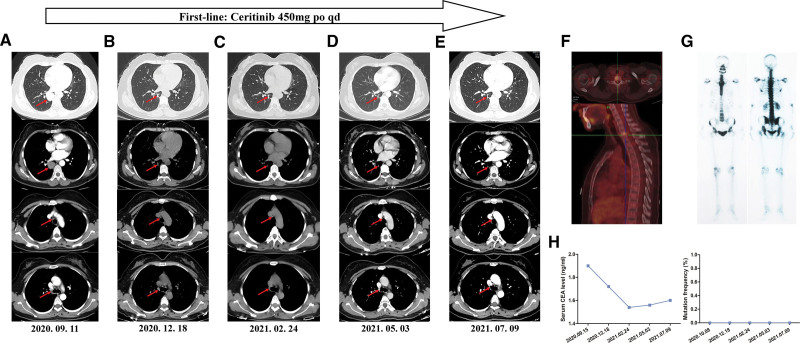
Dynamic monitoring of the response of the patient with lung adenocarcinoma to ceritinib. (A) Chest computed tomography (CT) scans on September 11, 2020 revealed the presence of a mass in the lower lobe of the right lung and enlargement of mediastinal and right hilar lymph nodes. Significant (December 18, 2020) (B) and consistent (February 24, May 3 and July 9, 2021) (C–E) reduction of the tumor volume and enlargement of mediastinal/right hilar lymph nodes were observed by the follow-up CT scans after the first-line therapy with ceritinib. (F) Positron emission tomography computed tomography (PET-CT) scanning on September 17, 2020 revealed bone metastasis at the C7 vertebral body. (G) Emission computed tomographic (ECT) bone scanning after treatment with ceritinib for 6 months showed bone metastasis disappeared. (H) Dynamic change of serum carcinoembryonic antigen (CEA) level and intergenic region ALK mutation frequency in peripheral blood ctDNA during the treatment course.

Baseline lymph node biopsy specimens and plasma samples were immediately subjected to comprehensive genomic profiling using NGS. Although targeted NGS of circulating tumor DNA (ctDNA) identified no aberrations in the EGFR, ROS-1, MET, HER-2, RET, or BRAF genes, we found a rare SLC8A1 downstream intergenic region ALK fusion at a 43.3% mutant allele frequency in the node biopsy specimen sample (Fig. 2A and B) which was not found in peripheral blood (Fig. 3H). CtDNA analysis revealed atypical fusion. In addition, ALK fusion was further validated at the mRNA level by real-time quantitative polymerase chain reaction (qPCR) (Fig. 2C), and immunohistochemistry (IHC) was performed to confirm ALK protein expression (Fig. 2D). Results from the phase 3 ASCEND-4 study demonstrated consistent, high, and durable antitumor efficacy of ceritinib in patients with advanced ALK-rearranged NSCLC.^[5]^ On the molecular findings, the patient underwent ceritinib therapy (450mg orally once daily), which ameliorated her clinical symptoms significantly and promptly. Best overall response to treatment was determined according to RECIST version 1.1, which was assessed locally at each institution.

After 2 months, chest CT scan images obtained during ceritinib treatment demonstrated a significantly reduced tumor volume and smaller mediastinal/right hilar lymph nodes (Fig. 3B), and sustained response after nearly 9 months with the best evaluation of partial remission (PR) (Fig. 3C–E). Emission computed tomography (ECT) bone scanning 6 months after therapy showed no abnormalities, including the C7 vertebral body (Fig. 3G). We did not detect brain metastasis during the clinical process of diagnosis and treatment of this patient using PET-CT and brain magnetic resonance imaging (MRI) scanning. During ceritinib treatment, serum CEA levels were normal (Fig. 3H). We dynamically monitored the mutation frequency of SLC8A1-ALK in the ctDNA of the peripheral blood during several routine follow-up visits. Interestingly, consistent with the objective response of the right lower lobe lung mass and mediastinal/right hilar lymph nodes by imaging assessment, SLC8A1-ALK intergenic fusion could not be detected in peripheral blood (Fig. 3H), accurately reflecting the real-time tumor response to ceritinib therapy. Currently, the disease is stable, and the patient is still receiving ceritinib treatment with good tolerance.

## 3. Discussion

In recent years, ALK has been proven to be another driver oncogene accounting for 3–7% of all patients with NSCLC; it is more prevalent in young and nonsmoking patients.^[6,7]^ Approximately 5% of patients with lung adenocarcinoma present with ALK rearrangements, which define a distinct molecular subgroup of NSCLC.^[8]^ Nevertheless, in addition to the classic ALK fusion partner EML4, other emerging ALK fusion partners present great challenges to clinical therapy.^[9]^ Crizotinib, the first ALK inhibitor drug approved by the FDA, showed an outstanding response in patients with advanced NSCLC positive for ALK rearrangement. However, most patients treated with crizotinib ultimately progress.^[9,10]^ Ceritinib is a next-generation, selective oral ALK inhibitor with a 20 times greater potency than crizotinib in enzymatic assays, which leads to suppression of ALK phosphorylation.^[11]^ In 2017, the phase 3 ASCEND-4 study showed that NSCLC patients with ALK rearrangement could obtain outstanding survival benefits with first-line ceritinib therapy.^[5]^ In addition, the ASCEND-8 study demonstrated that once daily ceritinib dose of 450 mg taken with food presents a similar exposure as the approved dose of 750 mg fasted.^[12]^ Herein, we report a lung adenocarcinoma patient with a rare ALK fusion partner, the SLC8A1 downstream intergenic region, never previously reported in the PubMed database, and obtained a remarkable clinical benefit after ceritinib (450mg with food) treatment.

To the best of our knowledge, this is the first report of breakpoints in the IGR downstream of SLC8A1 and within the ALK gene, as well as a novel ALK rearrangement. This fusion gene retains the complete ALK domain, which is a critical region for ALK activity. To guide ALK inhibitor therapy in patients with NSCLC, qPCR and IHC were used to identify ALK fusion status. However, previous studies have shown that tumor response to ALK inhibitors is heterogeneous in patients with ALK-positive NSCLC. One explanation for this confusing phenomenon is that diverse ALK fusion variants may result in disparate clinical outcomes.^[13,14]^

To the best of our knowledge, the shortcomings of traditional qPCR and IHC methods are that the precise ALK fusion variants cannot be identified, and the application of NGS could be used as an important and optional method.^[15]^ During ceritinib therapy, dynamic monitoring of the mutation frequency of the ALK rearrangement gene in peripheral blood by NGS was conducted. Interestingly, the mutation frequency of the ALK rearrangement gene was not always found during ceritinib treatment, which is consistent with the CEA level in the peripheral blood. Therefore, dynamic monitoring of the mutation frequency of ALK rearrangement might be a promising method for determining prognosis during therapy with an ALK inhibitor. The disease stabilized after the patient was treated with ceritinib for approximately 9 months. We will continue to follow up this patient. The patient’s remarkable response to ceritinib has expanded the spectrum of ALK fusions and provides useful information for precise ALK inhibitor administration in the future.

## 4. Conclusion

We present the first case of a rare SLC8A1 downstream intergenic region of ALK fusion in an advanced lung adenocarcinoma patient treated effectively with ceritinib using powerful NGS. However, whether this finding represents an inherent property of this fusion protein or its unique clinicopathological characteristics in patients with this fusion remains to be investigated. Moreover, the patient’s durable response to ceritinib and future resistance mechanisms require further follow-up.

## Author contributions

Conceptualization: Yanbei Zhang, Lei Fang, Guozheng Ding.

Data curation: Lei Fang, Guozheng Ding, Yuanzi Ye.

Funding acquisition: Yuanzi Ye.

Investigation: Lei Fang, Guozheng Ding, Muzi Wang, Peishan Ding.

Supervision: Yanbei Zhang, Lei Fang.

Validation: Yanbei Zhang, Lei Fang, Guozheng Ding.

Writing—original draft: Lei Fang.

Writing—review & editing: Xuebo Yan, Jiong Wang.
